# Influence of a lifestyle intervention in preschool children on physiological and psychological parameters (Ballabeina): study design of a cluster randomized controlled trial

**DOI:** 10.1186/1471-2458-9-94

**Published:** 2009-03-31

**Authors:** Iris Niederer, Susi Kriemler, Lukas Zahner, Flavia Bürgi, Vincent Ebenegger, Tim Hartmann, Ursina Meyer, Christian Schindler, Andreas Nydegger, Pedro Marques-Vidal, Jardena J Puder

**Affiliations:** 1 Institute of Exercise and Health Sciences, University of Basel, Basel, Switzerland; 2 Institute of Sports Sciences and Physical Education, University of Lausanne, Lausanne, Switzerland; 3 Institute of Social and Preventive Medicine, University of Basel, Basel, Switzerland; 4 Department of Paediatric Gastroenterology and Nutrition, Centre Hospitalier Universitaire Vaudois, University of Lausanne, Lausanne, Switzerland; 5 Institute of Social and Preventive Medicine, Centre Hospitalier Universitaire Vaudois, University of Lausanne, Lausanne, Switzerland; 6 Service of Endocrinology, Diabetes and Metabolism, Centre Hospitalier Universitaire Vaudois, University of Lausanne, Lausanne, Switzerland

## Abstract

**Background:**

Childhood obesity and physical inactivity are increasing dramatically worldwide. Children of low socioeconomic status and/or children of migrant background are especially at risk. In general, the overall effectiveness of school-based programs on health-related outcomes has been disappointing. A special gap exists for younger children and in high risk groups.

**Methods/Design:**

This paper describes the rationale, design, curriculum, and evaluation of a multicenter preschool randomized intervention study conducted in areas with a high migrant population in two out of 26 Swiss cantons. Twenty preschool classes in the German (canton St. Gallen) and another 20 in the French (canton Vaud) part of Switzerland were separately selected and randomized to an intervention and a control arm by the use of opaque envelopes. The multidisciplinary lifestyle intervention aimed to increase physical activity and sleep duration, to reinforce healthy nutrition and eating behaviour, and to reduce media use. According to the ecological model, it included children, their parents and the teachers. The regular teachers performed the majority of the intervention and were supported by a local health promoter. The intervention included physical activity lessons, adaptation of the built infrastructure; promotion of regional extracurricular physical activity; playful lessons about nutrition, media use and sleep, funny homework cards and information materials for teachers and parents. It lasted one school year. Baseline and post-intervention evaluations were performed in both arms. Primary outcome measures included BMI and aerobic fitness (20 m shuttle run test). Secondary outcomes included total (skinfolds, bioelectrical impedance) and central (waist circumference) body fat, motor abilities (obstacle course, static and dynamic balance), physical activity and sleep duration (accelerometry and questionnaires), nutritional behaviour and food intake, media use, quality of life and signs of hyperactivity (questionnaires), attention and spatial working memory ability (two validated tests). Researchers were blinded to group allocation.

**Discussion:**

The purpose of this paper is to outline the design of a school-based multicenter cluster randomized, controlled trial aiming to reduce body mass index and to increase aerobic fitness in preschool children in culturally different parts of Switzerland with a high migrant population.

**Trial Registration:**

Trial Registration: clinicaltrials.gov NCT00674544

## Background

Obesity is considered to be a global epidemic by the World Health Organization [1]. The marked increase in childhood obesity is alarming and already present in preschool children reaching 26% in 2- to 5-year old children and to 37% in 6- to 11-year old children [2]. In Switzerland there is a prevalence of overweight and obesity of around 20% and 23% in 6- to 12-year old boys and girls [3]. The prevalence of overweight/obesity and of physical inactivity is especially increased in children of low socioeconomic status (SES) [4] and/or children of migrant background [5,6].

Obese children are at increased risk to become obese adults [7,8] and this tracking becomes stronger the closer the child gets to adult status [9]. Yet, overweight preschool children have an over fivefold risk to be overweight at age twelve years compared with normal weight preschoolers [8]. Childhood obesity is already associated with cardiovascular disease risk factors [10-13] as well as other complications [7,8] and is an independent predictor of coronary heart disease in adulthood [14].

The main environmental causes attributed to the enormous increase in body fatness in the last few decades are an increase in energy intake through food and a decrease in energy expenditure through a decrease in physical activity (PA) and/or an increase in sedentary behaviour [15]. One of the most important contributors to sedentary behaviour is media use (TV, PC, game use) [16] which is also related to energy intake [16]. Another postulated factor associated with obesity and insulin resistance is a lack of sleep [17]. Social, cultural and economic factors also influence energy balance.

In the last years, cross-sectional and longitudinal data have shown that the increased intake of foods with high fat or sugar content [18], high energy snacks, sweets and sugar-added beverages is associated with increasing BMI [18-20]. In addition, over the last 20 years, aerobic fitness has decreased by around 8% in children from developed countries [21]. In contrast to aerobic fitness, there are no population-level objective data on temporal changes in total PA. However, some data indicate that children have become less physically active or less engaged in sports participation in the last years [22,23]. Nowadays, 3- to 5-year old children monitored with accelerometers spent around 80% of their time in activities of <1100 counts/min [24], which is considered to be sedentary behaviour or at most light PA [25]. In children, physical inactivity and reduced aerobic fitness are associated with increasing prevalence of cardiovascular risk factors [26-28] even independently of weight status [28,29].

As the great majority of obesity treatment studies show a lack of selected and longstanding effectiveness [30], primary prevention is absolutely essential. But short- and long-term studies in recent reviews show only small or no positive effects in BMI, SF and/or PA [31-34]. Implementing successful studies or projects is even more difficult in children from families of less advantaged SES and/or migrant background [34,35]. Although the period between the ages four and seven (the timing and the magnitude of the so called obesity rebound) has been suggested as a crucial time for development of overweight and obesity in children, there is a lack of studies in younger children [34]. For these reasons, we developed a study to assess the effect of a multidisciplinary lifestyle intervention on BMI and aerobic fitness by focusing on a young age group (preschoolers) and on children of migrant background (Ballabeina – Kinder im Gleichgewicht/enfants en équilibre). Ballabeina is Rhaeto-Romanic and means swing, teeter-totter, seesaw. This name of the study stands for a life in drive but also in balance.

### Theoretical model

**Figure 1:**
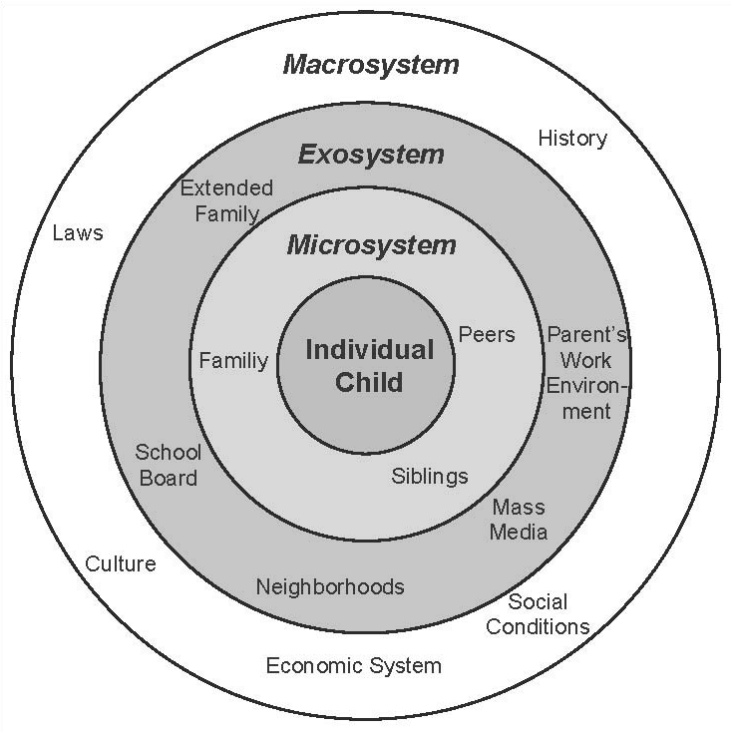
**Ecological Model**. Bronfenbrenner's Ecological Model describing the environmental influences on a child, with permission from [39].

Causes for overweight and obesity are multifaceted and prevention is difficult and complex. In the last years, social models of health promotion have been increasingly used to study complex interactions [36,37], as simple interventions are unlikely to work on their own and the development of effective preventive interventions requires strategies that affect multiple settings simultaneously [38]. Ballabeina is based on the social ecological model [36] (figure 1), that includes concentric rings that influence lifestyle patterns. The "psychobiologic core" of the model represents the genetic, physiologic, and socio-cultural forces that shape ones identity (**individual child**). This core is surrounded by the **microsystem**, the immediate environments with which a child interacts (parents, siblings, teachers, peers, etc.). The **exosystem **includes environments with which the child doesn't usually directly interact, but that can still affect the child (school boards, etc.). The **macrosystem **includes the broad societal settings under which the other cycles function (culture, history, social norms, economic system, etc.). For preschooler, the two main influence factors are the family and the teachers [39]. That's why the main intervention targets included these settings. The program promotes a healthy lifestyle by positively influencing personal, behavioral, and environmental factors. On the one side the intervention program transferred knowledge about adequate PA, nutrition and healthy food selection, reduced media use and proper sleep. On the other side the intervention also seeked to change the behavior of the child by increasing skills like motor abilities and augmenting daily PA. In addition the children and parents learned in a practical way strategies to change their nutritional behavior according to five nutrition messages (see below). The teachers achieved competencies by implementing PA and nutrition lessons in the preschool. On the environmental level, the built infrastructure (in- and outdoor in preschool) was adapted to enhance the child's natural behaviour to move and to explore. Participation of the children in extracurricular sport activities (club, etc.) in their neighbourhoods was promoted. The Ballabeina team also collaborated with the school boards, the building authorities and the school health services.

## Methods/Design

### Study objectives

The aim of the study was to evaluate the effects of a multidisciplinary multilevel lifestyle intervention in preschool children (aged 4- to 6-years) during one school-year in a multicenter cluster randomized controlled trial. The study included 40 randomly selected preschool classes and was conducted in the French (canton Vaud, VD) and in the German (canton St. Gallen, SG) part of Switzerland, focusing on areas with a high prevalence of migrant children.

### Main outcomes

Primary outcomes:

• BMI

• Aerobic fitness (20 m shuttle run)

Secondary outcomes:

• Total (sum of four SF, bioelectrical impedance) and central (waist circumference) body fatness

• Other motor abilities (obstacle course, balance platform, balance beam)

• PA and sleep duration (accelerometry and questionnaires), media use, nutritional behaviour and food intake of the child and the family (all questionnaires)

• General health (child and family), health-related quality of life, presence of hyperactivity (all questionnaires)

• Cognition tests (testing attention and spatial working memory ability)

Null-hypothesis: Potential differences in the primary outcomes between the INT and the CON groups at the end of the intervention will be entirely due to chance.

### Study Design

**Figure 2:**
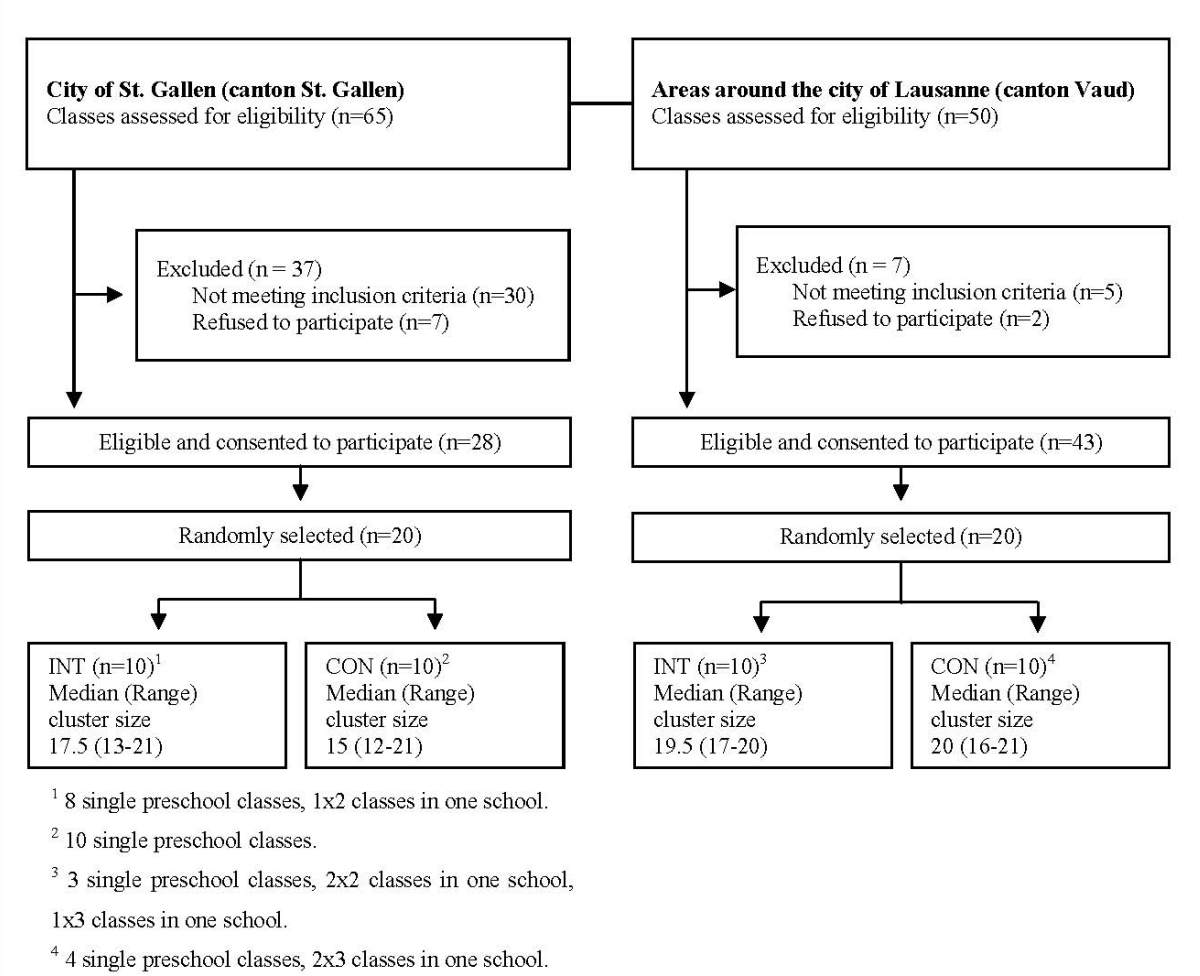
**Participants flow chart**.

Figure 2 shows a flow diagram of the recruited population. It was performed in two (SG & VD) out of 26 Swiss cantons. The German (SG) and the French (VD) parts of Switzerland represent two culturally distinct regions with different school and preschool systems. Classes from SG and VD were therefore separately selected and randomized after agreement of the school directors and the school health services of both cantons. The city of St. Gallen and the Lausanne area were chosen due to a high prevalence (i.e. at least 40%) of children of migrant background. Migrant background was defined as at least one parent born out of Switzerland. The prevalence of 40% was chosen as the school board estimated that in large adjacent areas with a high prevalence of a migrant population, 40–70% of children were of migrant background. For the selection and randomization opaque envelopes were used. For practical reasons, and to reduce an effect of contamination, preschool classes integrated in the same school building were randomized into the same group.

For all children an informed consent from a parent or a legal representative was necessary in order to participate in the study. Of the 727 children visited the chosen preschools, consent was obtained from parents/legal representatives for 655 (343 in the INT and 312 in the CON, participation rate: 90.1%).

The study was approved by the cantonal ethical committees of St. Gallen and Vaud.

### Need assessment, preplanning and pilot studies

In a first step a broad state of the art of health promoting projects in Swiss preschools [40-44] and a requirement analysis (knowledge, existing offers and barriers) was done. Teachers, health professionals and migrant experts were interviewed and asked to respond to a structured questionnaire. We also interviewed parents of migrant background with special emphasis on nutrition and PA behaviours (Jörg R, unpublished license of diploma, University of Basel). We performed qualitative interviews and designed and distributed questionnaires about their health perception, individual needs and attitudes towards offers in five preschool classes. Physical education classes were visited and analyzed. Based on this analysis, we determined content and transmission of information, as well as the extracurricular offers. We then performed different pilot studies (table 1) before the beginning of the main intervention.

**Table 1:** Overview of the different pilot studies:

**Pilot studies evaluating the intervention (PI)**	**Pilot studies evaluating the measurements (PM)**
11/2006: Testing of 10 PA home activity cards in 5 preschool classes for 4 weeks. Evaluation by teachers and parents (questionnaires).	

5/2007: Testing of 2 further PA and 2 nutrition home activity cards in 4 preschool classes. Evaluation by teachers and parents (questionnaires).	

11/2007: Testing of daily PA and weekly nutritional lessons with their home activity cards during 3 weeks in 1 preschool class. Evaluation by teachers and parents (questionnaires).	Feasibility and selection of tests 11/2007: Evaluation of 10 motor ability tests in one preschool class and selection of 4 of them by a team of sports scientists. Evaluation of the anthropometric measurements and the cognitive tests.

	Feasibility and reliability of tests 4/2008: Evaluation of the 4 motor ability tests in 2 preschool classes. Test-retest reliability of the balance platform test (static balance) and the anthropometric measurements.

	Reliability of motor ability tests 6/2008: Test-retest reliability of the "obstacle course" test (combined motor ability) and the balance beam test (dynamic balance) 1 preschool class.

### Intervention

The intervention was developed with input from exercise physiologists, preschool and primary school teachers, paediatricians, dieticians, psychologists and various stakeholders including experts for migrant families). The intervention focused on four topics: PA, nutrition, media use and sleep duration and was primarily applied at the level of the teachers, children and parents. All INT classes proceeded according to the same curriculum i.e. workshops, lessons, home activities, offers of extra-curricular activities, adaptation of the built infrastructure. The teachers were coached by trained health promoters (HP). These were physical education teachers who were further trained by a dietician and a physician. Theses HP intervened on the level of the teachers, the children, the parents and the local community. The CON group continued to follow their usual school curriculum which included one 45 min physical education lesson taught by the classroom teachers and one 45 min rhythmic lesson (given by a rhythmic specialist) for the French part of the study. Children were blinded to the existence of INT classes. The teachers and the parents, however, knew about the intervention arm. Participants, parents and school personnel, including classroom teachers, were informed that the intervention would promote their children's health, but were unaware of the main objectives.

#### Teachers

Prior to the intervention, the teachers took part in two afternoon workshops on the four topics (PA, nutrition, media use and sleep). In these workshops the teachers learned how to work with the lessons, the homework cards, the new PA infrastructure material. During the study, regular informal exchanges between the teachers and the HP took place and two formal meetings were organized.

#### Children

##### PA lessons

**Figure 3:**
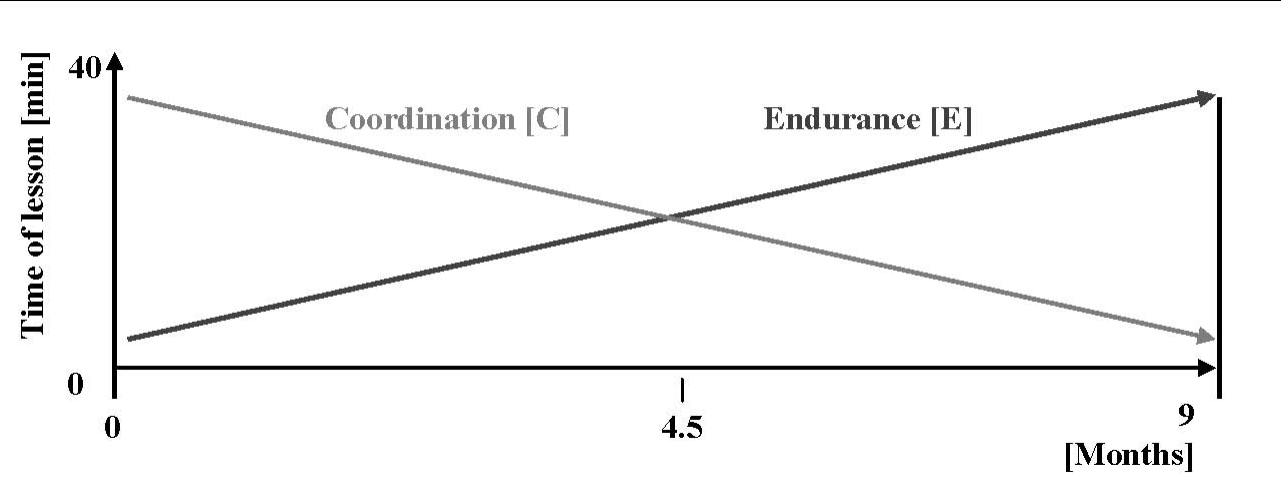
**Model for the physical lessons**. The physical activity lessons focused on coordination and endurance, but their distribution within one 40 min lesson changed over the period of the study.

PA lessons were given four times a week including 40 min lessons and 5 min cool down. In the beginning, one of the four lessons was given by the HP with the regular classroom teachers attending these lessons. After four months of intervention, the HP reduced their contribution to twice a month while the remaining lessons were taken over by the preschool teachers. All PA lessons were prepared by an exercise physiologist. The lessons took place in or around the preschool and once a week in the gym. Training of coordination and endurance was performed as described in figure 3. Additional sports equipment for the lessons such as balls, skipping ropes was offered and organized. Adherence to the PA lessons was assessed by regular classroom teachers.

##### Nutrition, media use and sleep lessons

The intervention on healthy balanced nutrition included weekly 45 min nutrition lessons, where the children learned balanced nutrition and healthy nutritional behaviour in a playful way. These lessons were developed and prepared by a dietician. The lessons were based on five messages i.e. "drink water", "eat fruit and vegetables", "eat regularly", "make clever choices", "turn your screen off when you eat" [42] that were transmitted in the form of a nutrition disk, developed in collaboration with the Swiss Society for Nutrition [45]. Each message was taught during a two-weeks period and was presented in two cycles over the year. Each message was described on a funny card which was taken at home with a task to implement the message at home (see below). During two additional weeks, lessons about sleep were implemented.

##### Infrastructural changes

The infrastructures of the preschool were adapted, in coordination with the building department to ensure the insurance guidelines for prevention of accidents.

##### Extracurricular activities

This included an additional weekly PA lesson (e.g. clubs). Where there was no offer of inexpensive all-round weekly PA lessons a weekly extracurricular lesson from a new national PA program [46] was offered.

#### Children and parents

##### PA and nutrition home activities

Sixteen PA and five nutrition cards were developed by professional PA teachers/nutritionists in collaboration with Health Promotion Switzerland [43]. The children got every other week a new PA or a nutrition activity card at home. These cards include specific PA tasks to be done at home. Some of these activities focused on a team work, which should promote the integration of other family members. A text on the backside of the card included simple information and practical hints to the parents. A CD with specific music for most PA cards was created to increase pleasure and define the minimal time the activity should be performed.

##### Events

Toward the end of the intervention, a morning event was organized, where children and parents participated together in a fun program while implementing the main messages of the study.

#### Parents

##### Information evenings

The HP organized two information evenings in each preschool. During the first information evening the HP informed about the study, the intervention, the testing and the informed consent. This information evening was performed also for the CON classes. On the second information evening, the HP presented the nutrition disk, informed about the five nutrition and media use messages and discussed possibilities and barriers of implementation. A third information evening performed by a dietician discussed the possibility of healthy nutrition that is cheap, tasty and can be easily and rapidly prepared.

##### Information booklet

In a short booklet, parents got informed about: (1) details of the intervention (2) practical hints to increase PA for children and for adults (3) existing PA offers for preschooler in the neighbourhood (4) the material, the children need at home for the home activities (5) the nutrition disc and (6) recommendations for a healthy mid-morning snack.

With the second information event the parents received the nutrition disc (offered in ten languages) and two other booklets about cooking and eating („Gemeinsam Kochen und Essen“, Cleven-Becker-Stiftung, 2008) and about PA in daily life ("Bewegung ist Leben", Bundesamt für Sport BASPO, 2008), offered in eight languages.

### Measurements

Measurements at baseline and at the end of the intervention were accomplished during a time period of five weeks (beginning both times in SG). Table 2 gives an overview about all measurements taken. The measurements were collected in three teams: anthropometry/concentration/memory (tested in the preschool class), motor abilities (tested in the gym) and PA (accelerometry). These teams worked parallel in different classes. With few exceptions the local teams did not change between the two testing periods. The main investigators for SG and VD were trained together to minimize inter-observer variability. Research assistants were blinded to group allocation. If a child was sick, BMI was measured few days later and questionnaires were distributed.

**Table 2:** Overview of the measurements:

Anthropometry and body composition	PA, nutritional intake and behaviour, media use, sleep duration
Height	Accelerometers, questionnaires*
Weight	Food frequency questionnaire*
Waist and hip circumference	
Skinfold thickness (triceps, biceps, subscapular,	Psychosocial health
suprailiacal)	General health of the child and the family*
Bioelectrical impedance (4-Polar)	Health-related quality of life (HRQOL)*
	Signs of Hyperactivity (SDQ)*
Motor ability	
Shuttle run test (aerobic fitness)	Cognition tests
Balance platform (static balance)	Attention (KHV-VK)
Balance beam (dynamic balance)	Spatial working memory ability (IDS)
Obstacle course (combination)	

* evaluated by a total of two questionnaires (one for lifestyle parameters, general and psychosocial health, one food frequency questionnaire)

#### Anthropometry and body composition

Standing height was determined and body weight was measured using an electronic scale (Seca, Basel, Switzerland; accuracy 0.05 g). Waist (midway between the iliac crest and the lowest border of the rib cage) and hip circumference (at the largest circumference) were measured by a flexible tape. SF thickness was measured in triplicate to the nearest 0.5 mm with Harpenden calipers (HSK-BI, British Indicators, UK) calibrated to exert a pressure of 10 g/cm^2 ^to the skin. Four sites (triceps, biceps, subscapular and suprailiac) were measured based on standard procedures [47]. The same four investigators took all measurements. Percent body fat was calculated according to the formulas of Slaughter, Deurenberg and Dezenberg [48-50] validated in preschool children [51,52]. The calculation of % body fat with this method has a prediction error of 3–5% [48,49]. The intra- and interobserver correlations in the pilot study (n = 21) using Spearman rank correlation analyses were r = 0.95 (p < 0.001) and r = 0.90 (p < 0.001), respectively for waist circumference and r = 0.98 (p = 0.001) and r = 0.96 (p = 0.001), respectively for the sum of four SF. Bioelectrical impedance was measured by a 4-polar single frequency device (RJL Systems, Model 101A; Detroit, MI, USA). The unit was calibrated prior to each testing day using a 500 ohm resistor provided by the company. Measurements were taken based on standard procedure[53]. If the distance from the proximal to the distal electrode was less than 5 cm in small children, the proximal electrode was located more proximal until the distance of 5 cm was attained. Percent body fat was calculated based on validated formulas [52,54,55]. The coefficient of variation between different bioelectrical impedance analysis measurements was less than 1.5% and for the calculation of fat-free mass it was 5.8% [52].

#### Motor abilities

##### Shuttle run test

The maximal multistage 20 m shuttle run test (20-MST) was used to assess aerobic fitness [56]. The test measures aerobic capacity by running forth and back for 20 m with an initial running speed of 8.0 km/h and a progressive 0.5 km/h increase of the running speed every minute that is indicated by a sound. The maximal performance was determined when the child was twice in series more than 3 m behind the given time or the child decided itself to stop because of exhaustion. The 20-MST has been found to be reliable (test-retest r = 0.73–0.93) [56-58], a valid measure of maximum oxygen consumption as measured by treadmill testing (r = 0.69–0.87) [57-61], and sensitive to changes in 6- to 16-year old children [61]. Some formal adoptions were made due to the very young age of the children by marking tracks on the floor to prevent the children from running curves and by an adult running with the children to provide the adequate pace.

##### Obstacle course

This test of overall fitness includes running 1 m from a marking cone to a transversally positioned bench, jumping over the bench (36 cm high, 28 cm wide), underpass this bench and running back to the marking cone three times in a row as fast as possible. Time was measured in seconds. This test was described by Vogt (1978) [62] and Kunz (1993) [63] as an ideal test for 3- to 6-year old children to test the overall fitness. The bench was constructed according to the German DIN 7909 standard except for the stabilizing bar, which was left out. Each child had two attempts and the faster one was used. Tests were considered invalid according to predefined criteria, i.e. if the performance was obviously submaximal, or the child did a mistake. The intra- and interobserver correlations in the pilot study (n = 14) using Spearman rank correlation analyses were r = 0.99 (p < 0.01) and r = 0.82 (p < 0.01), respectively. Less than 10% of the children had one invalid attempt and none had two invalid attempts.

##### Balance platform

Static postural control was measured in accordance to a standardized protocol [64] on a balance platform (GKS 1000^®^, IMM, Mittweida, Germany). The balance platform consisted of four sensors measuring displacements of the center of pressure (COP) in medio-lateral and anterior-posterior direction. Data acquisition was monitored (40 Hz) for 25 sec [64]. A balance-pad (Airex balance Pad, Airex, Aalen-Ebnat, Germany) was put on the balance platform, increasing the difficulty of the test. Postural sway was collected measuring the displacement of the COP. The smallest total length of two trial was used for further analysis. For experimental testing, children were asked to stand barefoot, with a 2 cm distance between both heels and feet placed in a 30° angle on the balance-pad, where coloured foot prints were placed. Hands were placed on the hips. After a test-stand for five seconds and a break while children descended from the force plate, the two trials were collected. The intraobserver test-retest correlation for the total length between the two attempts in the pilot study (n = 40) using Spearman rank correlation analyses was r = 0.73 (p < 0.0001).

##### Balance beam

According to Keogh (1965) balance beams are a suitable tool for testing dynamic balance in children [65]. In pilot testing we observed that balancing backwards was too difficult for children aged 4- to 6-years but balancing forward on a 3 cm wide balance beam was feasible and discriminated between children with high and low motor skills. We therefore included balancing barefoot forward on a 3 m long and 3 cm wide balance beam. The number of successful steps on the beam were counted until the child's foot touched the floor. Children performed three trials. The mean of the best two trials was calculated and used for further analyses. The intra- and interobserver correlation between the two better attempts in a pilot study (n = 15) using Spearman rank correlation analyses were r = 0.84 (p < 0.01) and r = 0.97 (p < 0.01), respectively.

#### Physical activity

PA was assessed by an accelerometer (MTI/CSA 7164, Actigraph, Shalimar, FL, USA). The accelerometers were constantly worn around the hip over five days at baseline and at the end of the intervention (both summertime) in the INT and in the CON group. The sampling epoch was set at 15 sec. This instrument has been shown to be valid across different activities in 3- to 5-year old children with a Pearson correlation coefficient between VO_2 _(ml/kg per min) and Actigraph counts/15 sec of r = 0.82 [66].

#### Questionnaires

Table 3 gives an overview of the two used questionnaires [67-74]. The reliability of a semi-qualitative food frequency questionnaire was tested in three classes assessing nutritional behaviour and food intake of preschool children of predominantly migrant background (Ebenegger, V. manuscript in preparation). Items were chosen from different food frequency questionnaires [67-69] adapted to the Swiss situation and the age group. This food frequency questionnaire was also filled in for each sibling aged two years or older.

**Table 3:** Overview of the questionnaires:

General Health Questionnaire	Food frequency questionnaire (adapted from [67-69])
• PA and participation in sports clubs of the child and the family [73]	• Nutritional behavior (i.e. if and where (i.e. home, day care) the meals were eaten, eating while watching television, eating alone)
• media use and sleep duration [74] of the child and its siblings	• Intake of 15 different categories of food during the last 4 weeks (subdivided into nutriments)
• General health of the family members	
• Parental height and weight	
• Socioeconomic data (i.e. education, origin, nationality and cultural integration)	
• health-related quality of life (HRQOL)* [70] • presence of a hyperactive behavior with the Strengths and Difficulties Questionnaire (SDQ)** [71]	

* HRQOL was measured by the German version of the PedsQL 4.0TM (Pediatric Quality of Life Inventory) Generic Core Scales (U.S. Copyright Registration No. TXu 856-101) with a parent proxy-report, containing four scales (Physical, Emotional, Social, School) and 23 items. The psychometric properties of the PedsQL 4.0TM justify application in a healthy child population [70]. ** The presence of hyperactive behaviour was evaluated with the Strengths and Difficulties Questionnaire (SDQ) [71]. The parent proxy-form comprised the hyperactivity/inattention scale consisting of five items. Validity has been demonstrated in healthy children and adolescents [72].

#### Cognition tests

To measure attention ability, the *Konzentrations-Handlungsverfahren für Vorschulkinder *(KHV-VK) [75] was applied. Test material consists of 44 cards with familiar pictures, which had to be sorted into four different boxes. Sorting time and error quote allowed quantitative and qualitative statements on attention. The test has been validated in a preschool population and age specific norms are available. Test-retest reliability was r = 0.88 [75].

Spatial working memory ability was measured by a subtest taken from the Intelligence and Development Scales (IDS) [76]. Thereby geometrical forms had to be memorized and identified. Significant correlations to related measures confirmed construct validity (i.e. HAWIK-IV Working memory scale: r = 0.52) and the test-retest reliability was r = 0.48 [77,78].

### Evaluation

All evaluation measures were developed as defined in the CONSORT guidelines [79]. We will evaluate the intervention with regard to primary and secondary outcome measures. We will also perform a process evaluation to assess the appreciation the feasibility and the subjective effectiveness of the program by teachers (questionnaires and semi-qualitative interviews) and parents (questionnaires).

### Data analysis

Baseline comparability of INT and CON schools will be assessed using descriptive statistics and two sample *t *test for continuous and χ^2 ^test for categorical variables. If necessary, variables will be logarithmically transformed before analyses. As a primary prevention program, the intervention was designed to target the entire sample. Effects are expected and intended to occur throughout the entire distribution of adiposity and aerobic fitness in the sample – not just around a defined threshold. Thus, for purposes of establishing the efficacy of this intervention, it is most appropriate to compare the full distributions of BMI and aerobic fitness between INT and CON groups. Therefore, to test the primary hypotheses, accounting for the design with classes as the unit of randomization, mixed linear models will be used, with change in BMI and aerobic fitness as the dependent variable, study arm as the factor of interest and age, sex, language region (German vs. French part of Switzerland) and baseline BMI or aerobic fitness, respectively, as covariates. The same analytic approach will also be used for all secondary outcome variables. Potential interactions of intervention with sex or age will be tested for each outcome. Data will be analyzed according to intention to treat.

With an average class size of 18, we assumed that 13 children per class would participate in both shuttle run-tests (due to non-participation, attrition, moving, sickness on the testing day). A total number of 40 classes would then provide enough power to detect a true intervention effect of half an inter-subject standard deviation at the usual significance level of 0.05 with a probability of 0.9, provided that the standard deviation of the random class effect does not exceed 25% of the inter-subject standard deviation (i.e., corresponding to an intra-class correlation of about 0.06).

The following subgroups will be also investigated: Normal weight and overweight/obese children, children with low baseline fitness, children with migrant background and Swiss children, children of low socioeconomic background.

## Discussion

We achieve to develop a concise and appropriate protocol for the development and implementation of a multilevel lifestyle intervention aiming to prevent weight gain and to increase aerobic fitness in a high-risk preschool population with a high percentage of migrant background. We believe that the inclusion of stakeholders such as teachers, parents and school directors from the very beginning, the extended preplanning inlcuding testing and evaluation of the intervention material and the theory-driven multilevel approach will improve the likelihood of a successful intervention.

The purpose of this paper was to outline the design of a multicomponent multilevel school-based multicenter cluster-randomized, lifestyle intervention trial aiming to reduce BMI and to increase aerobic fitness in 4- to 6-year old preschool children in culturally different parts of Switzerland with a high prevalence of migrant children. We aim to offer information and a solid base for a further adaption and larger implementation of prevention programs focusing on preschool children that are adapted to children of low SES and migrant background. Results of the intervention will be available in 2010.

## Competing interests

The authors declare that they have no competing interests.

## Authors' contributions

JJP, SK and LZ designed the study. JJP was the principal investigator and is guarantor. JJP, SK, IN, FB, VE, AN, TH and PM established the methods and questionnaires. IN, FB, VE and JP were the main coordinators of the study. IN, FB, VE, UM, AN, PM and JJP conducted the study. CS and PM gave statistical and epidemiological support. IN wrote the article under the assistance of JJP and got additional help from SK and PM. JJP obtained the funding, with the assistance of SK and LZ. All authors provided comments on the drafts and have read and approved the final version.

## Pre-publication history

The pre-publication history for this paper can be accessed here:


